# Belatacept and carfilzomib-based treatment for antibody-mediated rejection in a sensitized nonhuman primate kidney transplantation model

**DOI:** 10.3389/frtra.2023.1230393

**Published:** 2023-09-01

**Authors:** Robin Schmitz, Miriam Manook, Zachary Fitch, Imran Anwar, Isabel DeLaura, Danae Olaso, Ashley Choi, Janghoon Yoon, Yeeun Bae, Mingqing Song, Alton B. Farris, Jean Kwun, Stuart Knechtle

**Affiliations:** ^1^ Department of Surgery, Duke Transplant Center, Duke University Medical Center, Durham, NC, United States; ^2^ Department of Pathology, Emory University School of Medicine, Atlanta, GA, United States

**Keywords:** belatacept, carfilzomib, antibody-mediated rejection, allosensitization, nonhuman primate

## Abstract

**Introduction:**

One-third of HLA-incompatible kidney transplant recipients experience antibody mediated rejection (AMR) with limited treatment options. This study describes a novel treatment strategy for AMR consisting of proteasome inhibition and costimulation blockade with or without complement inhibition in a nonhuman primate model of kidney transplantation.

**Methods:**

All rhesus macaques in the present study were sensitized to maximally MHC-mismatched donors by two sequential skin transplants prior to kidney transplant from the same donor. All primates received induction therapy with rhesus-specific ATG (rhATG) and were maintained on various immunosuppressive regimens. Primates were monitored postoperatively for signs of acute AMR, which was defined as worsening kidney function resistant to high dose steroid rescue therapy, and a rise in serum donor-specific antibody (DSA) levels. Kidney biopsies were performed to confirm AMR using Banff criteria. AMR treatment consisted of carfilzomib and belatacept for a maximum of four weeks with or without complement inhibitor.

**Results:**

Treatment with carfilzomib and belatacept was well tolerated and no treatment-specific side effects were observed. After initiation of treatment, we observed a reduction of class I and class II DSA in all primates. Most importantly, primates had improved kidney function evident by reduced serum creatinine and BUN as well as increased urine output. A four-week treatment was able to extend graft survival by up to two months.

**Discussion:**

In summary, combined carfilzomib and belatacept effectively treated AMR in our highly sensitized nonhuman primate model, resulting in normalization of renal function and prolonged allograft survival. This regimen may translate into clinical practice to improve outcomes of patients experiencing AMR.

## Introduction

Despite advances in immunosuppressive drugs, antibody-mediated rejection (AMR) remains a critical problem in solid organ transplantation: 30% of transplant recipients of all organs develop AMR at some point after transplantation (1, 2). While current standard of care (SOC) immunosuppressive regimens target T cell-mediated rejection (TCMR), there is currently no reliable and durable treatment for AMR. As AMR is mediated by donor-specific antibody (DSA), the most widely-used approaches aim to rapidly remove DSA and to eliminate antibody-producing cells (e.g., B cells and plasma cells) but none durably modulate antibody production. Such approaches include plasmapheresis (PP), intravenous immunoglobulin (IVIg), anti-CD20mAb, increased maintenance immunosuppression, and/or lymphocyte-depleting agents (3–6). IVIg and PP are considered first-line treatments for AMR despite lack of evidence of efficacy or safety (4, 7). Furthermore, no mechanistic evidence suggests that approaches targeting antibodies alone can adequately control DSA production in the long term.

Rituximab, an anti-CD20 mAb, is the third most commonly used agent for AMR treatment and its use in clinical practice has increased as initial or rescue therapy (4, 8–10). As with antibody-targeting approaches, evidence for the efficacy of rituximab in treating AMR is limited (11). A single-center study of rituximab with PP for treatment of AMR reported two-year graft survival rates at 90% in the dual treatment group compared to 60% in the PP alone group (12). In contrast, multi-center clinical trials evaluating rituximab and standard of care (SOC: plasma exchange, IVIg, and corticosteroids) or rituximab and IVIg found no difference in graft survival, renal function, or adverse events (13, 14).

As such, there are currently no FDA-approved treatment for AMR. A recent meeting of international experts noted that there was no conclusive evidence to support any specific AMR therapies, highlighting the need to develop and rigorously evaluate AMR therapies (15).

Many biologics are available to target different components of the humoral response (16). These agents target initial B cell help from T cells (costimulation blockade) (17, 18), B cell survival (BAFF) (19), plasma cells (proteasome inhibitor, anti-CD38mAb) (17, 18, 20–22), antibodies (i.e., Imlifidase or anti-FcRn mAb) (23–25) and complement activation or split products (i.e., Eculizumab, C1-INH) (26–28), and have been tested in the clinic or in pre-clinical models for a variety of indications. However, optimal outcomes may not be achieved by just targeting a single component of the humoral response, due to compensatory pathways and mechanisms (20). We hypothesized that combination therapies offer a more promising approach to modulate the post-transplant humoral immune response.

Approaches to AMR treatment are often similar to those used for desensitization therapies prior to HLA-incompatible transplants, given the similar pathophysiologic mechanisms. Indeed, desensitization therapies aim to reduce the immune barrier by removing antibodies and targeting antibody-producing cells. Accordingly, therapies that successfully achieve this goal in the context of desensitization provide promising candidates for the treatment of AMR. Our group has previously tested several dual desensitization strategies of costimulation blockade with proteasome inhibition in sensitized NHP (17, 18, 29, 30). The combination of belatacept and carfilzomib effectively reduced plasma cell and follicular helper T cell (Tfh) populations, which reduced the incidence of early AMR and prolonged kidney allograft survival (29). Therefore, we hypothesized that proteasome inhibition and costimulation blockade with carfilzomib and belatacept would reduce antibody-mediated graft damage and prolonging graft survival in the context of AMR.

## Materials and methods

### Animal selection and AMR treatment

All animals (Macaca mulatta) were sensitized with serial skin transplantations or multiple pregnancies and received a life-sustaining kidney allograft from maximally MAMU-mismatched donors as previously reported (17, 29, 31). All primates received induction therapy with rhesus-specific ATG (rhATG) and were maintained on different immunosuppressive regimens. Twelve (12) animals [but not three (3) animals in acute AMR group] received pre-transplant desensitization as shown in Table 1. A total of fifteen (*n* = 15) rhesus macaques who developed AMR (based on graft dysfunction with sCr > 2 mg/dl, DSA elevation, and histopathology) were included for analysis in the present study. All animals with clinical signs of AMR were first treated with corticosteroids (125 mg/kg daily for 3 days). Twelve (12) animals were additionally treated with belatacept (20 mg/kg, IV) and carfilzomib (27 mg/m^2^, IV) weekly for 4 weeks with or without the C3 complement inhibitor, Compstatin (Cp40, 2 mg/kg TID) for 2 weeks (Table 1). Animals in this report have appeared in previous publications with respect to data unrelated to AMR treatment (28, 32–34). In other words, treatment of AMR and response to treatment data have not previously been reported. For all surgical procedures, animals were treated with pre-operative ketamine (10–30 mg/kg, IM) for induction and intra-operative isoflurane (1.5–5%) for the entire operation for anesthesia. For Euthanasia, Euthasol at 1 ml/10pound (lb) body weight was administered IV following initial sedation with ketamine at 10–30 mg/kg IM. All medications and procedures were conducted in accordance with the Duke Division of Lab Animal Resources (DLAR) and the National Institutes of Health (Bethesda, MD) guidelines after approval by Duke University (Durham, NC) Institutional Animal Care and Use Committees (IACUC #A153-18-06 or #A113-21-05).

**Table 1:** Treatment, immunosuppression, and graft survival of NHP recipients.

Experimental group	ID	AMR diagnosis day	Pre-transplant desensitization	Immunosuppression at AMR	AMR treatment	Additional urvival (day)
Acute AMR control (*n* = 3)	HADV	7	None	Tac/MMF/Steroid	None	5
	H49G	7	None	Tac/MMF/Steroid	None	1
	H75W	7	None	Tac/MMF/Steroid	None	1
Late AMR control (*n* = 4)	H50R	96	Lulizumab/CFZ	Tac/MMF/Steroid	None	6
	H46G	48	Lulizumab/CFZ	Tac/MMF/Steroid	None	4
	H58G	88	Lulizumab/CFZ	Tac/MMF/Steroid	None	9
	H77A	60	Bela/CFZ	Tac/MMF/Steroid	None	11
Late AMR treated (*n* = 5)	H72E	16	Cp40	Tac/MMF/Steroid	Bela/CFZ/Cp40	10
	H77P	33	Cp40	Tac/MMF/Steroid	Bela/CFZ/Cp40	67
	H87T	46	Cp40	Tac/MMF/Steroid	Bela/CFZ/Cp40	21
	DR27	49	Bela/CFZ	Tac/MMF/Steroid	Bela/CFZ/Cp40	15
	DR7G	68	Bela/CFZ	Tac/MMF/Steroid	Bela/CFZ	140
Chronic AMR treated (*n* = 3)	J978	181	Bela/CFZ	Belatacept monotherapy	Bela/CFZ	51
	H99A	322	Bela/CFZ	No immunosuppression	Bela/CFZ	36
	J979	175	Bela/CFZ	Tac/MMF/Steroid	Bela/CFZ	371

### Flow cytometric crossmatch

Recipient serum samples were collected weekly throughout the study period. For flow cytometric crossmatch analysis, donor cells (from whole blood or spleen) were stained with viability dye (Fixable Blue, Invitrogen, Carsbad, CA) according to the manufacturer's manual. Cells were washed with PBS (2%FBS) and blocked with Goat IgG whole molecule (1:1000 dilution, Jackson ImmunoResearch Laboratory Inc., West Grove, PA). After 15 min of incubation at 4°C, cells (5 × 10^6^) were washed with PBS (2%PBS) and cocultured with diluted (1:50) recipient serum samples for 30 min at 4°C. After washing 5 times with PBS (2% FBS) and cells were then stained with PE-conjugated anti-CD20 (clone 2H7, BD Biosciences San Jose, CA), PerCPCy5.5-conjugated anti-CD3 (clone SP34-2, BD Biosciences), and FITC-conjugated polyclonal anti-monkey IgG (Seracare Life Science Inc. Milford, MA) for T cell fluorescent crossmatch (TFCX) and B cell fluorescent crossmatch (BFCX). Cells were washed twice with PBS (2% FBS) and fixed with Stabilizing Fixative (BD Biosciences). Flow cytometry was performed on a BD LSRFortessa flow cytometer and analyzed using FlowJo v9 or v10 software.

### Histological analysis

Kidney allograft and lymph node biopsies were performed before and after AMR treatment as well as at the time of euthanasia. Biopsy/necropsy specimens from kidney allografts were fixed in 10% neutral buffered formalin, paraffin embedded, sectioned, and stained with hematoxylin and eosin (H&E), periodic acid-Schiff (PAS), and polyclonal anti-human C4d (American Research Products, Waltham, MA) for histologic evaluation. Graft histology was evaluated in a blinded fashion by an experienced transplant pathologist (A.B.F) and scored according to the current Banff criteria for kidney rejection (35–38). Peripheral lymph node biopsies were collected from the axilla (axillary lymph nodes) or groin (inguinal lymph nodes).

### Statistical analysis

All data are expressed individually or as the mean ± SD (with error bar) throughout the manuscript. Statistical analyses were performed using GraphPad Prism 9.0 (GraphPad Software, San Diego, CA, USA). Values of *P* < 0.05 were considered to be statistically significant. Survival analysis was performed using the Kaplan–Meier method and log-rank test. Normally distributed data within the same treatment group but at different time points were evaluated using a paired *t*-test. Statistical comparisons between different groups were performed with unpaired *t*-test for normally distributed data.

## Results

### Belatacept and carfilzomib improve kidney allograft function and prolong graft survival in the setting of late AMR

**Figure 1:**
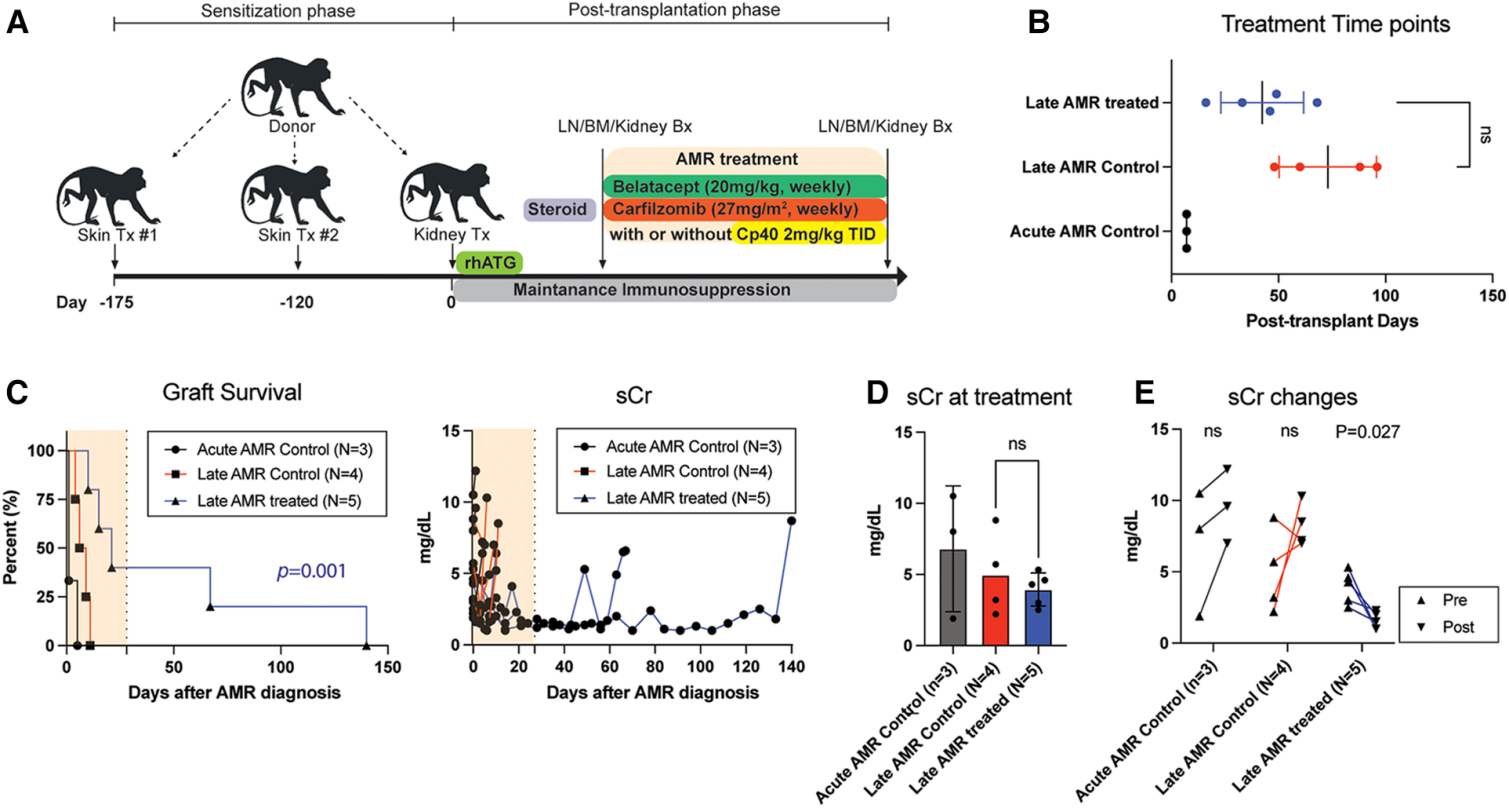
Belatacept and carfilzomib-based AMR treatment prolonged graft survival in sensitized kidney recipients. (**A**) Schematic representation of the allo-sensitization, AMR treatment regimen, and timing of the treatment. (**B**) AMR treatment time-point for individual animals. NS indicates no statistical significance using two-tailed parametric unpaired *t*-test. (**C**) Graft survival after AMR diagnosis and treatment. AMR treatment (*n* = 5) significantly prolonged graft survival in sensitized recipients developing AMR compared to untreated control (*n* = 4). Survival analysis was performed using the Kaplan–Meier method and log-rank tests. (**D**) Serum creatinine (sCr) levels at AMR development. There was no difference between treatment vs. control groups. NS indicates no statistical significance using two-tailed parametric unpaired *t*-test. (**E**) Collated data for sCr changes after AMR development and treatment. Animals with AMR treatment showed significant reduction of sCr. *P*-values less than 0.05 are considered significant; ns indicates no statistical significance using two-tailed parametric paired *t*-test. All data are presented as individual values. N number indicates biologically independent animals.

In order to evaluate the efficacy of our novel AMR treatment regimen, primates with evidence of AMR were assigned to either undergo treatment with carfilzomib and belatacept or continues treatment with corticosteroids (Figure 1 and Table 1). Animals exhibiting rejection within the first week post-transplant were excluded due to the therapeutic window being too short to enact treatment. Therefore, we treated animals who developed AMR >14 days after kidney transplantation (Figure 1B). Upon development of AMR, five (5) animals were treated with weekly carfilzomib (27 mg/m^2^) and belatacept (20 mg/kg) IV for four weeks, and four (4) of these animals also received the C3 complement inhibitor Compstatin (Cp40, 2 mg/kg, TID, SC) for 2 weeks in order to provide immediate protection from DSA. The specific treatment criteria for these animals were (1) on-going worsening of kidney function following initially treatment with high dose corticosteroids; (2) presence of DSA. Four (4) control animals (late AMR controls) were solely treated with bolus corticosteroids. There was no significant difference between the onset of AMR and initiation of treatment between the two groups (Figure 1B). Two out of five treatment animals completed the 4-week treatment course, while two animals experienced graft failure while undergoing treatment and one animal was euthanized due to a chronic nonhealing wound with cardiac complication/infection complications. However, the median graft survival after intervention was significantly longer in the treatment group compared to the control group (21d vs. 7.5d, *P* = 0.01; Figure 1C). There was no significant difference of serum creatinine (sCr) level between the two groups at the start of AMR treatment (Figure 1D). Immediately after initiation of treatment, animals treated with carfilzomib and belatacept showed an improvement in sCr levels (Figure 1E). Of note, animals who received Compstatin in addition to carfilzomib/belatacept did not have any additional improvement in graft function or survival. Taken together, these data suggest that carfilzomib/belatacept inhibit AMR progression, leading to prolonged allograft survival.

### Belatacept/carfilzomib/Cp40 treatment reduces donor-specific antibody titers

**Figure 2:**
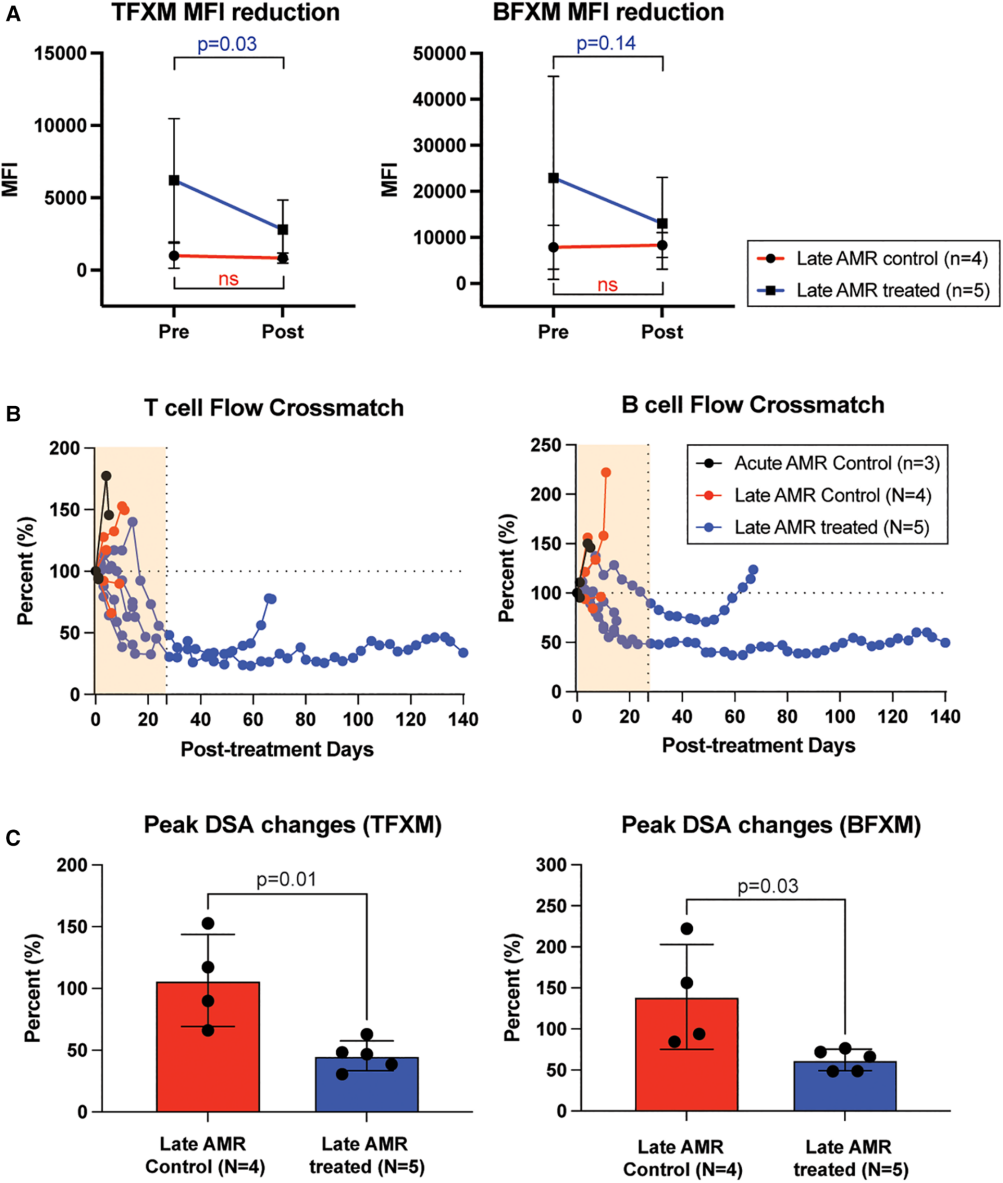
Belatacept and carfilzomib-based AMR treatment reversed serum DSA level. (**A**) The peak DSA changes after AMR diagnosis. DSA level was measured at the time of AMR diagnosis and at the time of euthanasia (for untreated control) or after the treatment (upto 1 month). (**B**) DSA kinetics after belatacept and carfilzomib-based AMR treatment. Post-AMR treatment DSA levels are normalized to the pre-treatment level. (**C**) DSA changes from individual animals. Animals treated with belatacept and carfilzomib-based AMR treatment showed higher level of reduction in TFXM and BFXM. All data are presented as mean ± S.D. N number indicates biologically-independent animals; *P*-values less than 0.05 are considered significant; NS indicates no statistical significance using two-tailed parametric unpaired *t*-test.

To evaluate whether the improved kidney function and prolonged graft survival in the treatment group were due to dampening of the humoral immune response, we measured serum DSA levels throughout the study period. Post-transplant DSA kinetics are shown in Figure 2. Congruent with their clinical course, serum DSA titers were not reduced in control animals following administration of high dose corticosteroids. In contrast, in animals receiving AMR treatment, T cell flow crossmatch (TFCM) showed a significant reduction of DSA after initiation of treatment while B cell flow crossmatch showed a trend towards reduced DSA titers (Figure 2A). However, both T and B cell crossmatch showed a significantly greater reduction of DSA in treated animals compared to control animals (Figure 2B). Of note, after completion of treatment, primates experienced a rebound of DSA. These data suggest that the combination of carfilzomib/belatacept/Cp40 can reverse the post-transplant humoral immune response in allosensitized primates after developing antibody-mediated rejection, leading to normalization of allograft function and prolonged survival.

### Antibody-mediated rejection at chronic stage is resistant to belatacept and carfilzomib treatment

**Figure 3:**
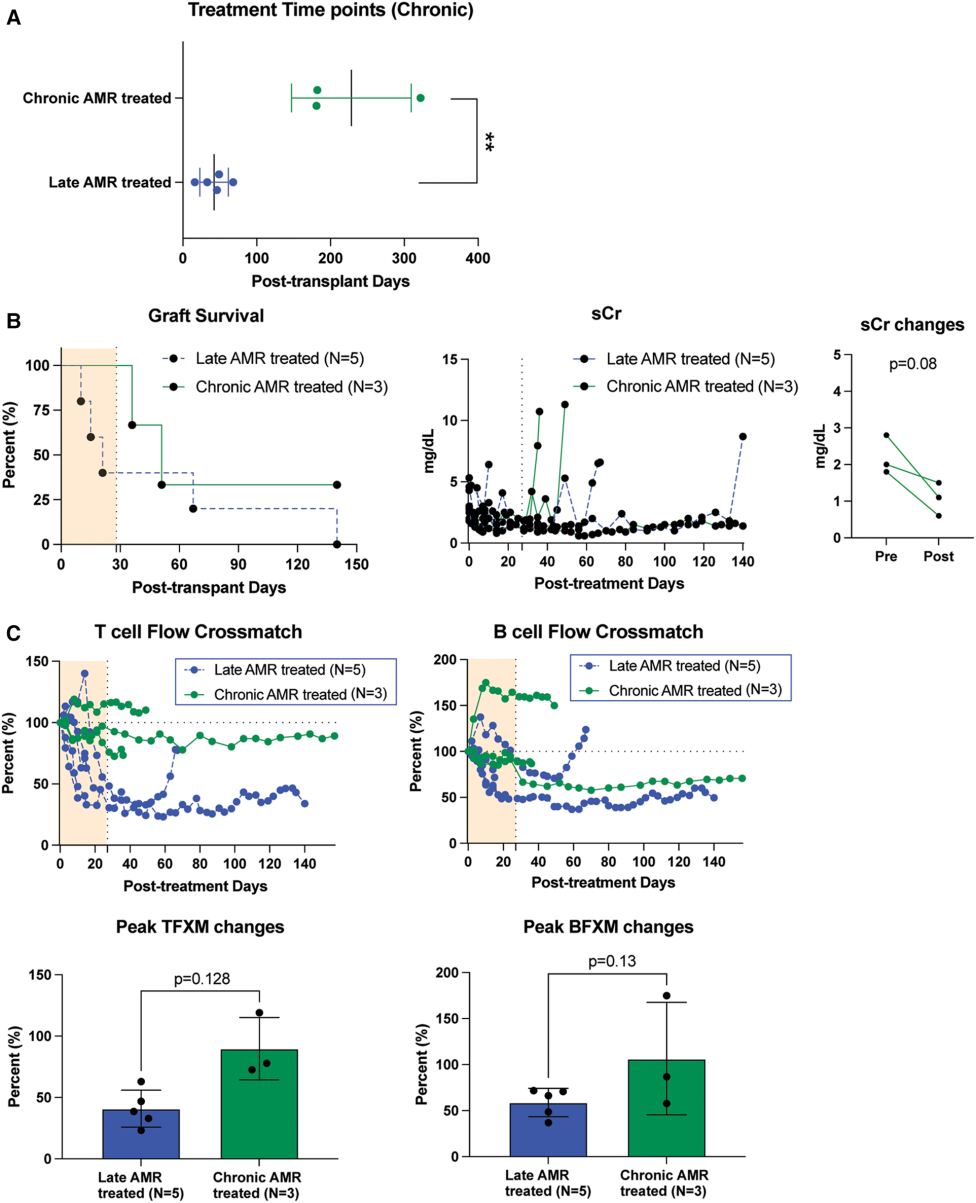
The impact of belatacept and carfilzomib-based AMR treatment on chronic AMR (>6 month post-transplantation. (**A**) Chronic AMR treated group developed AMR significantly later than late AMR group. (**B**) Graft survival after AMR diagnosis and treatment. AMR treatment for animals developed AMR > 6month post-transplantation (*n* = 3) showed similarly prolonged graft survival to late AMR treated group (*n* = 5). Survival analysis was performed using the Kaplan–Meier method and log-rank tests. (**C**) DSA changes from individual animals. TFXM and BFXM showed a trend of resistance of DSA reduction after the belatacept and carfilzomib-based AMR treatment in chronic AMR treated group. Post-AMR treatment DSA levels are normalized to the pre-treatment level. All data are presented as mean ± S.D. N number indicates biologically-independent animals; *P*-values less than 0.05 are considered significant using two-tailed parametric unpaired *t*-test.

**Figure 4:**
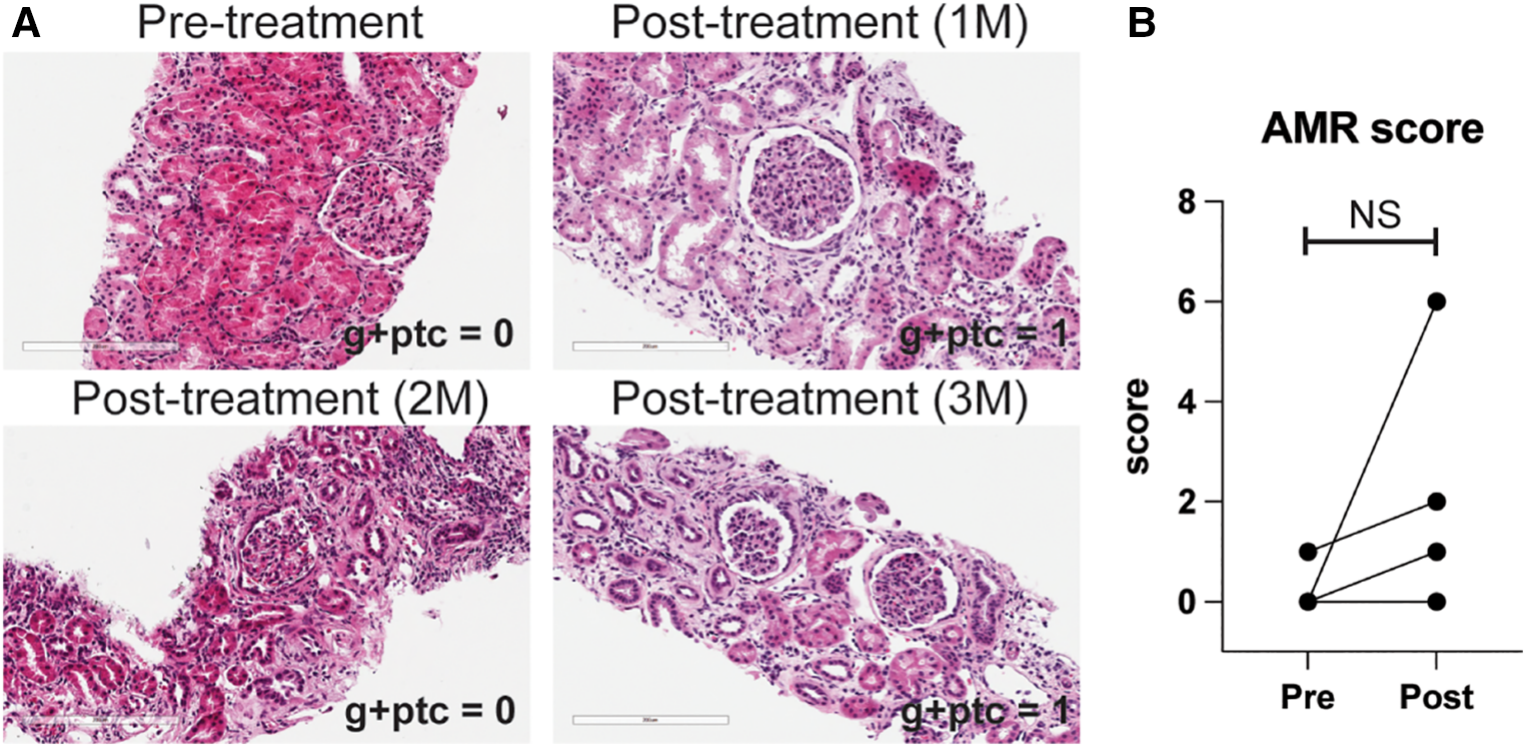
AMR pathology was not reversed by belatacept and carfilzomib-based treatment. (**A**) Representative H&E image of kidney biopsy before and after treatment. (**B**) AMR score (g + ptc) before and after the treatment in later AMR treated group (*n* = 4). All data are presented as mean ± S.D. N number indicates biologically-independent animals; *P*-values less than 0.05 are considered significant; NS indicates no statistical significance using two-tailed parametric paired *t*-test.

We also treated animals who developed antibody-mediated rejection at a much later time point >6 month after kidney transplantation. As shown in Figure 3A, these animals developed AMR (chronic AMR group) significantly later (226 ± 83.1d) than the late AMR group (42.4 ± 19.3d). Treated animals with chronic AMR (*n* = 3) showed similar graft survival to treated animals with late AMR (*n* = 5) (Figure 3B). Furthermore, all animals showed improved kidney function following treatment (Figure 3B). Despite this and in contrast to animals with late AMR, DSA titers were reduced less and at a slower rate (Figure 3C,D), suggesting that chronic AMR might be more treatment-resistant compared to late AMR. We performed serial kidney biopsies before and after belatacept and carfilzomib treatment to evaluate histologic changes throughout the treatment. Pathological gradings revealed no evidence of histologic reversal of AMR after treatment (Figure 4). As such, treatment appeared to prevent progression of AMR, rather than reverse AMR pathology, based on the prolonged survival with improved sCr levels.

## Discussion

This study presents the use of carfilzomib/belatacept therapy in a NHP model of AMR following kidney allotransplantation. Following development of graft dysfunction and corresponding increase in DSA, animals were treated with corticosteroids, carfilzomib, and belatacept, with or without the C3 complement inhibitor Compstatin, and compared to controls treated with corticosteroids alone (Figure 1). Treatment of AMR with carfilzomib and belatacept improved kidney allograft function, prolonged graft survival (Figure 1) and suppressed the production of DSA (Figure 2) in accordance with our previous study in which we established this regimen for pretransplant desensitization (29). The contribution of complement inhibition in this regimen needs to be further evaluated. Animals experiencing chronic AMR (as defined by onset > 6 months post-transplant) were less responsive to AMR treatment, with less reduction in DSA titers (Figure 3).

Our group has previously shown that dual desensitization with carfilzomib and belatacept effectively lowers DSA, plasma cells (PC), T follicular helper cells (Tfh), and B cell follicles in the germinal center (GC) (29). Prevention of cognate T-B cell interactions through costimulatory blockade inhibits GC reactions and prevents repopulation of PCs from alloreactive memory B cell (Bmem) reservoirs. Similar combination regimens such as carfilzomib with lulizumab or tocilizumab, and daratumumab and plerixafor have similarly blunted the post-transplant humoral response (21, 22, 30). Given similarities in desensitization and AMR treatments, we applied this dual targeting strategy to AMR. In particular, this study examines chronic AMR, which likely occurs in a unique immunological context compared to acute AMR. Acute AMR likely arises in the context of DSA whose formation has been initiated prior to treatment, produced by both PC and germinal center B cells. Furthermore, the plasma cell repertoire implicated in early AMR is likely comprised of short-lived PCs and plasmablasts compared to long-lived plasma cells in chronic AMR. Thus, the impact of costimulation blockade and proteasome inhibition is likely to be different in early acute vs. late chronic AMR as costimulatory/coinhibitory signals are implicated in GC activation and contraction, and proteasome inhibition has been shown to preferentially act upon plasmablasts (39). Accordingly, we observed decreased efficacy of carfilzomib and belatacept in the chronic AMR group.

The combination of proteasome inhibitor and costimulation blockade has been used in humans in treatment of acute post-kidney transplant AMR (40). Bortezomib/belatacept treatment was given after PP, IVIG, and steroids failed to improve graft function. This regimen successfully resolved the episodes of acute AMR in six kidney transplant recipients, and suppressed DSA for up to 30 months (40). While this combination did not reverse or permanently halt the process of AMR in any patient, it may constitute an improvement over current approaches to AMR treatment.

One major barrier to the development of effective AMR treatments is the lack of a clinically relevant and reproducible animal model. There is no existing model that consistently produces an AMR phenotype. Furthermore, since organ transplantation promotes intense responses from both T and B cells, we cannot selectively elicit AMR or TCMR in this outbred, large animal model. The timescale of rejection is also an important consideration. Clinically, chronic AMR is a major source of graft loss in kidney transplant recipients but in animal models the development of a chronic AMR phenotype is difficult to achieve practically, as it requires long-term humoral activation and production of DSA while avoiding graft loss from acute AMR and TCMR, infection, and animal welfare concerns. Furthermore, long-term maintenance of post-transplant NHPs is costly and difficult to justify without reproducibility of a chronic AMR phenotype. Accordingly, we used sensitized maximally-MHC mismatched NHP to accelerate the development of AMR—a process that takes years in human recipients. Maximal mismatching of kidney grafts is not a clinical goal since it results in greater risk of rejection and poorer graft survival; however, this approach experimentally may trigger a robust and accelerated AMR response, resulting in a stringent and practical model of AMR. Given this stringency, we hypothesize that patients undergoing HLA-compatible transplants may actually have better responses to AMR treatment compared to our NHPs. To address this limitation, we have recently developed a naturally sensitized multiparous female primate model (32). Additionally, all animals received desensitization therapy prior to transplant to avoid early acute AMR. The present study includes NHP that developed biopsy-proven AMR on late and chronic timescales while in other immunosuppression studies, thus providing a clinically relevant and resource-sparing approach to preclinical AMR models.

The inclusion of animals maintained on different immunosuppressive regimens is also a limitation. While the NHP were impartially assigned to control and treatment groups, we cannot account for the potential impact of varying immunosuppression on subsequent AMR treatment. Previous studies have shown that CNI-based regimens do not adequately control the humoral response and thus animals maintained on costimulation blockade-based regimens may respond better to carfilzomib and belatacept AMR therapy (41).

Despite these limitations, the present study provides a useful and resource-efficient preclinical model to develop AMR treatments. Belatacept and carfilzomib dual therapy for AMR resulted in significant prolongation of post-transplant survival via reduction of DSA production, but failed to prevent rebound under conventional CNI-based immunosuppression. Given the published clinical experiences and our preclinical data, carfilzomib and belatacept offer a promising approach to AMR treatment that may improve on SOC antibody-targeting strategies.

## Data Availability

The original contributions presented in the study are included in the article/Supplementary Material, further inquiries can be directed to the corresponding authors.
